# Comparing the effects of low-level laser therapy and gaseous ozone as a preventive measure on medication-related osteonecrosis of the jaws following tooth extraction: a rat model

**DOI:** 10.1186/s40001-024-01907-3

**Published:** 2024-07-09

**Authors:** Öznur Özalp, Oğuzcan Göksu, Havva Serap Toru, Mehmet Ali Altay, Alper Sindel

**Affiliations:** 1 Department of Oral and Maxillofacial Surgery, Faculty of Dentistry, Akdeniz University, Dumlupinar Boulevard, Campus, 07058 Antalya, Turkey; 2 Department of Pathology, Faculty of Medicine, Akdeniz University, Antalya, Turkey

**Keywords:** Bisphosphonate-associated osteonecrosis of the jaw, Low-level laser therapy, Osteonecrosis, Ozone

## Abstract

**Objectives:**

Use of numerous medications such as tyrosine kinase inhibitors (sunitinib), monoclonal antibodies (bevacizumab), fusion proteins (aflibercept), mTOR inhibitors (everolimus), radiopharmaceuticals (radium 223), selective estrogen receptor modulators (raloxifene), and immunosuppressants (methotrexate and corticosteroids) has been reported to be a risk factor for development of medication-related osteonecrosis of the jaws till date. This study aimed to evaluate the preventive effect of low-level laser therapy (LLLT) and gaseous ozone on the onset of MRONJ following tooth extraction.

**Materials and methods:**

A total of 40 male Wistar rats were randomly allocated into 4 groups of 10 rats each. The groups laser (L), ozone (O), and control (C) received weekly intraperitoneal injections of zoledronic acid (0.06 mg/kg), while group sham (S) received saline solution for 4 weeks. After the 4th injection, all subjects underwent mandibular first molar extraction and adjunctive laser or ozone was applied according to the groups. All the rats were sacrificed at 4 postoperative weeks for comparative histomorphometric evaluation of bone healing in extraction sites.

**Results:**

Laser and ozone groups demonstrated significantly higher bone formation compared to control group (*p* < 0.05), while no significant difference was found between laser and ozone groups (*p* = 1.00). Furthermore, the greatest bone formation was observed with the sham group (*p* < 0.05).

**Conclusions:**

Findings of the current study support that adjunctive LLLT and ozone therapy following tooth extraction may help prevent MRONJ and improve bone healing in subjects under zoledronic acid therapy.

**Clinical relevance:**

Since the introduction in 2003, great effort has been devoted to developing a certain management protocol for MRONJ. Several publications have appeared in recent years documenting promising results of adjunctive LLLT and ozone application in treatment of MRONJ. However, experimental data are limited on this regard and the present study, for the first time, aimed to evaluate and compare the effects of LLLT and ozone in prevention of MRONJ.

## Introduction

Medication-related osteonecrosis of the jaws (MRONJ) still presents a challenging complex disease with many questions remain unclear regarding its pathophysiology and proper management. A high incidence of drug-induced osteonecrosis of the jaw has been reported in patients using chronic and systemic antiresorptive drugs, mainly as a complication of surgical dental procedures or, in some cases, spontaneously. Till date, numerous families of medications such as tyrosine kinase inhibitors (sunitinib), monoclonal antibodies (bevacizumab), fusion proteins (aflibercept), mTOR inhibitors (everolimus), radiopharmaceuticals (radium 223), selective estrogen receptor modulators (raloxifene), and immunosuppressants (methotrexate and corticosteroids) have been implicated as risk factors for MRONJ [1, 2].

However, in the 2022 update of American Association of Oral and Maxillofacial Surgeons’ Position Paper on Medication-Related Osteonecrosis of the Jaws, only three main groups of antiresorptive drugs including bisphosphonates (BPs), denosumab and romosozumab have been reported to be associated with an increased risk for developing MRONJ [3]. Among these, BPs are the most famous major class of drugs for the treatment of various bone diseases [4]. BP therapy has proven to be one of the most effective ways to treat benign and malignant diseases characterized by high bone turnover, such as metastatic bone diseases, osteoporosis, Paget's disease, and pediatric osteogenesis imperfecta [5–7].

In the literature, there is no consensus on ideal management of MRONJ [8–10]. Treatment strategies mainly focus on minimizing the progression or formation of bone necrosis, eliminating pain, controlling infection, and optimizing the patient's quality of life [5, 8, 10]. For several years, great effort has been devoted on adjunctive therapies to prevent and manage MRONJ. Among them, several studies have reported promising results with the use of low-level laser therapy (LLLT) and ozone. It has been suggested that biostimulation through LLLT may be beneficial in MRONJ management to enhance bone healing by increasing osteoblast proliferation and differentiation, collagen type-I formation, and secretion of growth factors [11, 12].

In addition to LLLT, a few number of studies have been appeared recently claiming that ozone therapy may be utilized in MRONJ management due to its several biologic effects such as activation of neuroprotective systems, improvement of blood circulation and oxygen delivery, stimulation of immune modular system, and also prevention or treatment of infectious conditions due to its antibacterial, antiviral, and antifungal properties [13].

Despite this interest, experimental data are rather controversial and no one to the best of our knowledge has compared the effects of LLLT and ozone in prevention of MRONJ. The null hypothesis of the present study is local application of LLLT and gaseous ozone following tooth extraction have no positive effect on impaired bone healing under bisphosphonate medication. We therefore aimed to evaluate the preventive effect of low-level laser therapy and gaseous ozone on the onset of MRONJ following tooth extraction.

## Material and methods

### Animals and ethical approval

The regional animal research ethics committee (Akdeniz University, Antalya, Turkey) reviewed and approved the study protocol (B.30.2.AKD.0.05.07.00/97). All the procedures were carried out in compliance with the National Research Council’s Guide for the Care and Use of Laboratory Animals, the US Public Health Service’s Policy on Human Care and Use of Laboratory Animals, and the National Institutes of Health Guide for the Care and Use of Laboratory Animals.

### Study design

This study was performed on healthy adult male Wistar rats with a mean weight of 300 g obtained from Akdeniz University Experimental Animals Application and Research Center. The subjects were left for a 1-week study period to get used to the laboratory conditions before the experiment. Throughout the study, the subjects were placed in appropriate cages under the control of a veterinarian, with 2 subjects in each cage. All subjects were fed a standard laboratory diet (rat chow and tap water). All rats were housed in cages in triplicate at 22 ± 2 °C, at 40–60% humidity, in a constantly warm and fresh air, in a cycle of 12 h of light and 12 h of darkness.

The sample size was calculated using the "G. Power-3.1.9.2" program with a 95% confidence level [14]. The subjects were randomly allocated into 4 groups of 10 rats each (laser-L, ozone-O, control-C, and sham-S). The groups L, O, and C received weekly intraperitoneal injections of zoledronic acid (0.06 mg/kg) according to the protocol described by Zandi et al., while the group S received saline solution for 4 weeks [15].

**Fig. 1:**
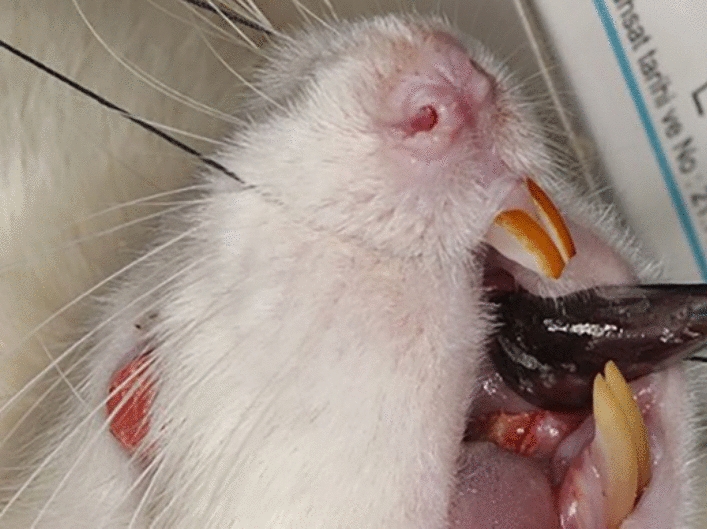
Mandibular first molar extraction was performed in all rats using periosteal elevators and forceps

One-week after the last zoledronate or saline injection, all rats underwent mandibular first molar extraction under general anesthesia using intraperitoneal injection of 90 mg/kg of ketamine hydrochloride (Ketalar^®^; Eczacibasi^®^, Istanbul, Turkey) and 20 mg/kg of xylazine (Alfazyne 2%; EgeVet, Izmir, Turkey). Tooth extraction was performed using a molt periosteal elevator to detach the gingiva and then a forceps to dislocate the tooth (Fig. 1).

**Fig. 2:**
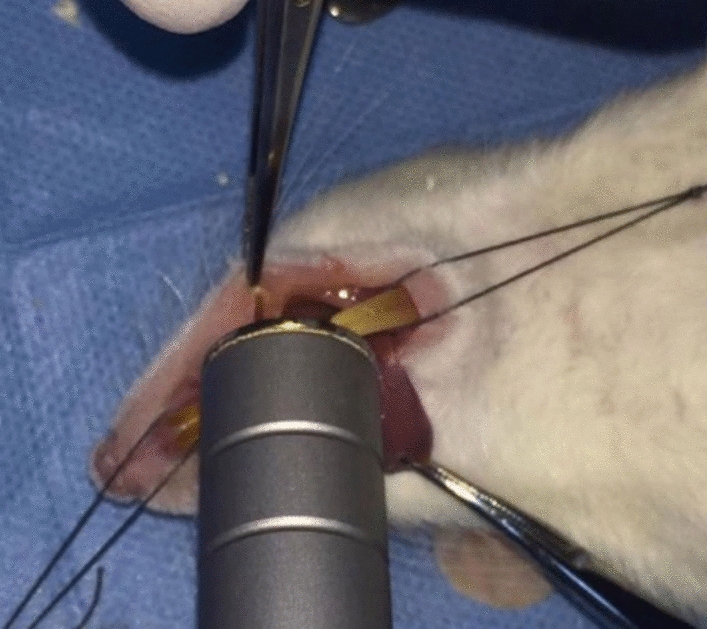
Ga-Al-As diode laser application following tooth extraction

Following the extraction, a gallium-aluminum-arsenide (Ga-Al-As) diode laser (Epic10; Biolase, Irvine, CA, USA) application performed to the 1st group (group L) with the following parameters: 808 nm wavelength, 0.5 W power, continuous wave, non-contact mode at 0.5–1 cm distance from the oral mucosa, spot size 0.28 cm^2^ (*R* = 6 mm), for 30 s, energy density of 5 J/cm^2^ (energy per point,1.4 J), and as completely cover the surgical area (Fig. 2). First application of laser was performed immediately after the surgery and repeated on 3rd, 5th, 7th, and 10th postoperative days [16].

**Fig. 3:**
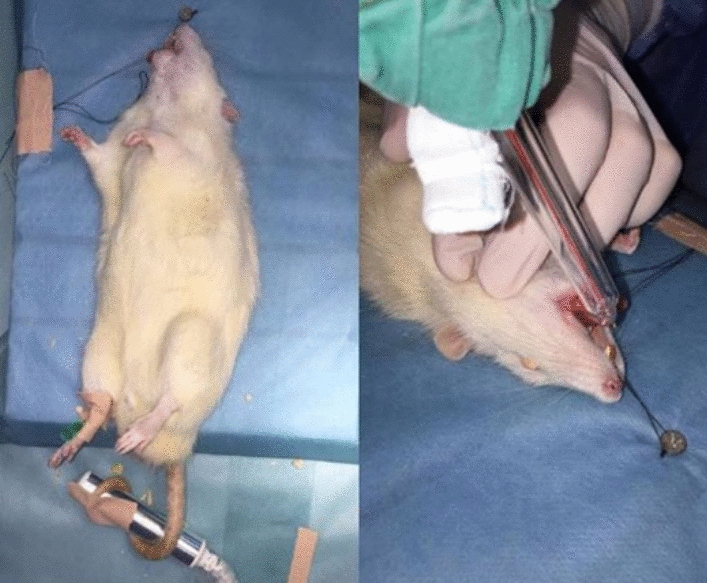
Ozone administration following tooth extraction

Ozone therapy was applied to the 2nd group (group O) using OzoneDTA device (OzoneDTA generator, APOZA, New Taipei, Taiwan) with 80% oxygen intensity for 30 s (Fig. 3). Similar to laser application, ozone was administered immediately after the surgery and repeated on 3rd, 5th, 7th, and 10th postoperative days [17].

**Fig. 4:**
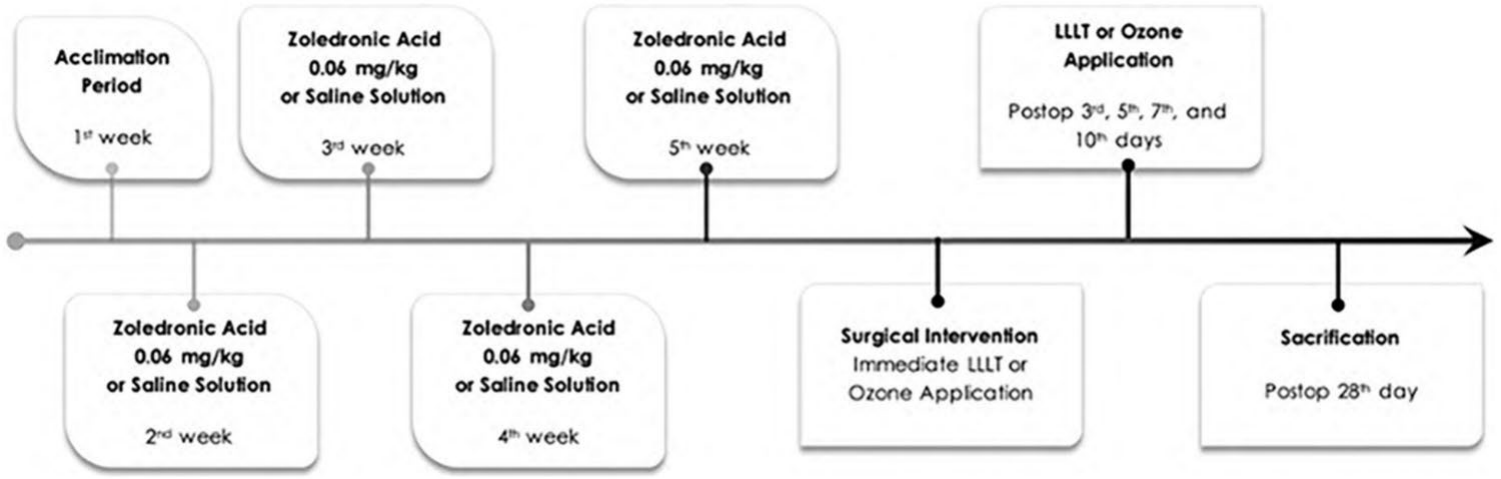
Timeline for the experimental design

Rats in control and sham groups did not receive any adjunctive therapy following the extraction. Postoperative medication included subcutaneous injection of 5 mg/kg of carprofen (Rimadyl; Pfizer, New York, NY) every 24 h for 5 days. At 4 postoperative weeks, the rats were sacrificed under general anesthesia by an intravenous (IV) overdose of thiopental sodium (150 mg/kg; Pental; IE Ulagay, Turkey). Figure 4 illustrates the timeline of the experimental design. Clinical examination was performed regarding the presence of bone exposure and intraoral/extraoral fistula.

### Histomorphometric analyses

**Fig. 5:**
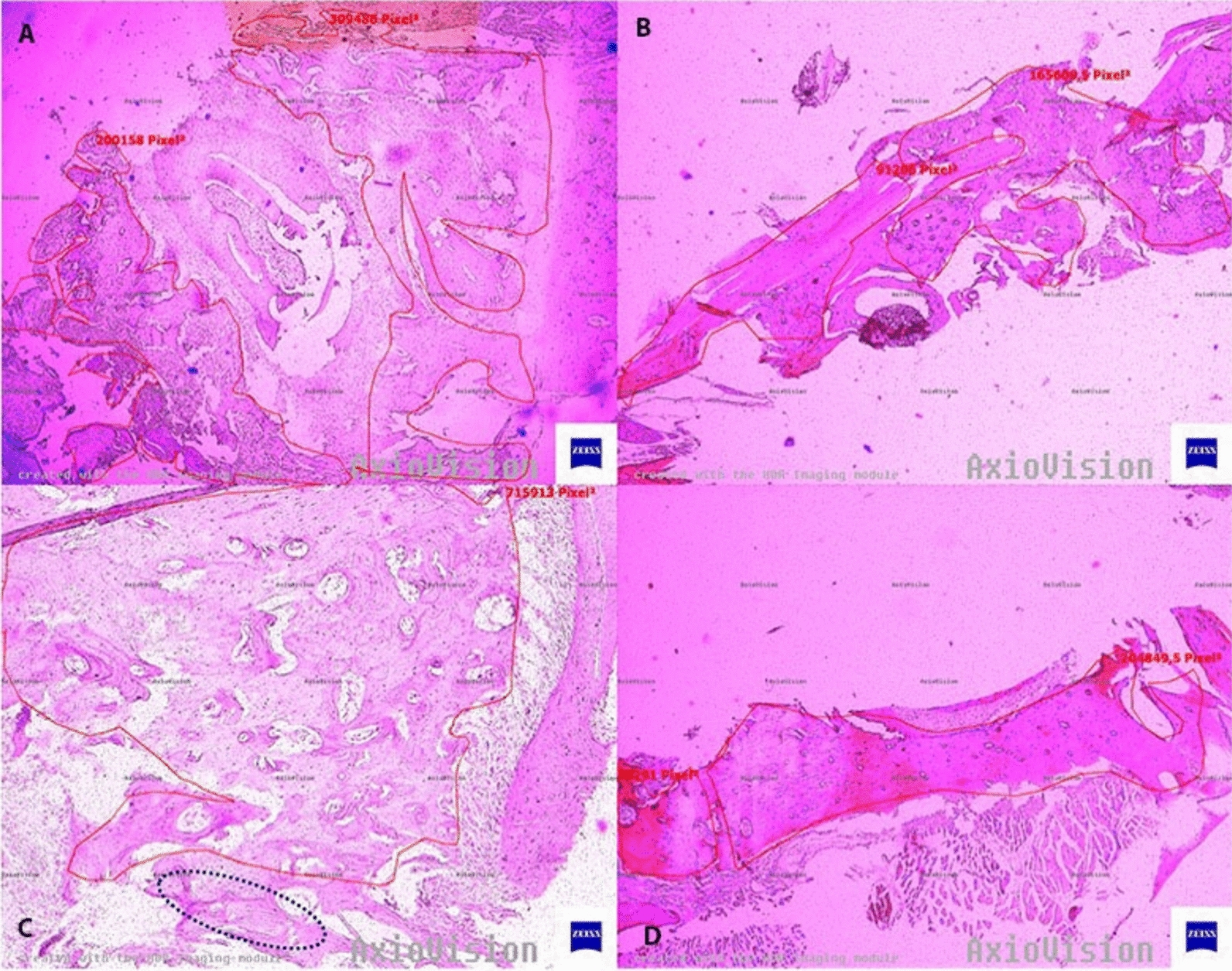
Histomorphometric measurement of new bone formation under × 4 magnification. **A** Sample from laser group; regular new bone formation without necrosis or inflammation. **B** Sample from ozone group; regular new bone formation without necrosis or inflammation. **C** Sample from control group; partial bone formation is observed, marked area represents bone necrosis with empty osteocyte lacunae. **D** Sample from sham group; intense new bone formation areas are observed with no sign of necrosis or inflammation

The hemi-mandibles of the 40 rats were excised and immediately immersed in 10% buffered formalin for 48 h and decalcified with 10% nitric acid and 10% sodium citrate for 3 days. After decalcification, the specimens were embedded in paraffin blocks. Sections of 6 µm thickness were cut in sagittal section along the long axis of the extraction socket using a slicer, stained with hematoxylin and eosin. All slides were examined under a light microscope (Axioscope 2 Plus; Carl Zeiss Microscopy LLC, New York, NY) connected to a computerized digital camera (AxioCam MRc; Carl Zeiss Microscopy GmbH, Gottingen, Germany). Subsequently, digital images were obtained with a microscopic camera (AxioCam MRc; Carl Zeiss Microscopy GmbH) at different magnification settings by a blinded pathologist. Following manual delineation on the images of slices, areas of new bone formation were measured automatically in square micrometers using a software program (AxioVision, release 4.8.2(06-2010); Carl Zeiss MicroImaging GmbH, Jena, Germany) (Fig. 5). The analysis was repeated three times and the mean values were assigned to each slide to be used for statistical analysis. For standardization of the samples, the same tooth (mandibular first molar) of rats of approximately same age and body weight was extracted and an identical total number of slices were obtained from each mandible.

### Statistical analysis

Descriptive statistics, including frequency, median, interquartile range, and percentage distributions, were used to analyze the data. Considering the small sample size, the Kruskal–Wallis test was performed, followed by Dunnett T3 post hoc analysis (differences were considered significant at *p* < 0.05) using IBM Corp. Released 2015. IBM SPSS Statistics for Macintosh, Version 23.0., Armonk, NY: IBM Corp.

## Results

**Fig. 6:**
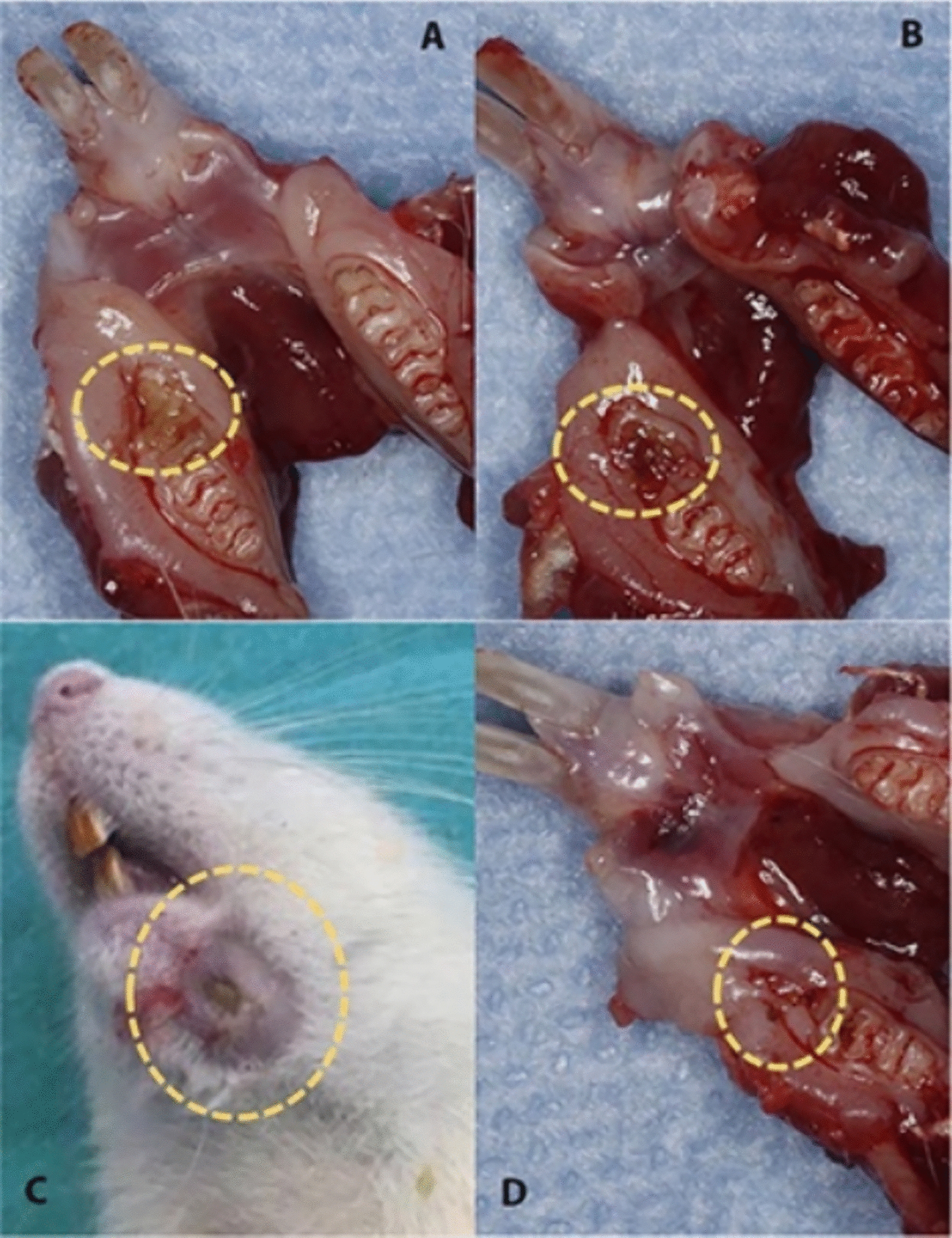
Gross clinical appearance for different groups at sacrification phase. **A** Laser group **B** Ozone group **C** Control group **D** Sham group

No rats were lost during the postoperative period. On clinical examination, the findings indicating impaired healing (e.g., mucosal ulceration, abscess, fistula formation, necrotic bone exposure) were found in 2 rats in group O (20%; *p* = 0.16), 3 in the group L (30%; *p* = 0.08), and 7 in the group C (70%; *p* = 0.00) (Fig. 6). Uneventful wound healing with intact overlying mucosa and no bone exposure was observed in all the rats in group S.

Statistically significant difference was observed between groups regarding bone formation (Table 1). Laser and ozone groups demonstrated significantly higher bone formation compared to control group (*p* < 0.05), while no significant difference was found between laser and ozone groups (*p* = 1.00) (Table 2). Furthermore, the greatest bone formation was observed with the sham group (*p* < 0.05) (Table 2).

**Table 1:** Kruskal–Wallis revealed significant difference (*) between groups regarding bone formation

N	40
Median	1093414,0000
Chi-Square	20,000
df	3
Asymp. Sig	0.000*

*N* number of subjects, *df* degree of freedom, *Asymp.Sig.* Asymptotic significance

**Table 2:** Dunnett 3 post hoc analysis for comparison of groups

Groups		Mean difference	Standard error	Significance
Ozone (O)	LLLT	−49497,30000	203416,07491	1.000
	Control	614899,00000^*^	175097,50494	0.022*
	Sham	−1252456,10000^*^	347692,12818	0.017*
LLLT (L)	Ozone	49497,30000	203416,07491	1.000
	Control	664396,30000^*^	146524,36752	0.003*
	sham	−1202958,80000^*^	334215,60413	0.020*
Control (C)	Ozone	−614899,00000^*^	175097,50494	0.022*
	LLLT	−664396,30000^*^	146524,36752	0.003*
	Sham	−1867355,10000^*^	317775,24565	0.001*
Sham (S)	Ozone	1252456,10000^*^	347692,12818	0.017*
	LLLT	1202958,80000^*^	334215,60413	0.020*
	Control	1867355,10000^*^	317775,24565	0.001*

(*) refers to significant difference

## Discussion

The objective of the present study was to compare for the first time the effects of adjunctive LLLT and ozone application following tooth extraction on prevention of MRONJ.

Although the pathogenesis of MRONJ is not yet clear, several studies have demonstrated that BPs reduce the expression of bone morphogenic protein-2 (BMP-2), which directly affects osteoblast differentiation and bone remodeling [18]. Furthermore, it has been shown that BP therapy increases the expression of transforming growth factor β1 (TGF-β1) which also plays an important role in bone remodeling through enhanced osteoblast differentiation [19]. An increase in TGF-β1 also results with reduced expression of nuclear factor kappa-B ligand (RANKL), which stimulates osteoclasts via its receptor [20].

As a result of above-mentioned and the other multiple pathways, BPs trigger alteration in the ratio of osteoblasts to osteoclasts in bone remodeling, leading to a reduction in bone resorption and turnover and finally accumulation of non-renewed and hypermineralized bone. One other potential mechanism of MRONJ is delayed wound healing due to impaired function of keratinocytes and fibroblasts resulting from BP-induced soft tissue toxicity [21].

In this regard, the present study aimed to evaluate and compare, in an experimental design, the effects of two different adjunctive therapies that recently gained interest, in both prevention and management of MRONJ.

In the molecular basis, it has been shown that LLLT might improve bone remodeling by stimulating the osseous integration with a high exposure of osteocalcin (OCN) and BMP-2 [22]. Moreover, several studies demonstrated that LLLT might prevent MRONJ by suppressing tissue inflammation via up-regulating interleukin-1 receptor antagonist (IL-1RA) expression, down-regulating interleukin-1β (IL-1β), tumor necrosis factor-α (TNF-α), and interleukin-6 (IL-6) in the gingival tissue, which results in an increase in collagen formation and early gingival wound healing [23–25].

LLLT stimulates cell proliferation and bone formation through the induction of cell cycle-regulating proteins [26, 27]. LLLT has potential antibacterial and biostimulator effects when applied to oral tissues [28]. Currently, laser biostimulation (or biomodulation) has a wide range of applications, primarily in the recovery and management of pain. Most studies on biostimulation of bone use visible and infrared diode lasers [29]. These observations suggest that laser biostimulation may aid in treatment of MRONJ. In a study by Altay et al., 11 patients with BRONJ underwent medical and surgical treatment supported with LLLT [14]. The authors reported that BRONJ lesions can effectively and safely be treated when the surgical procedure is performed in an atraumatic manner, under high doses of antibiotic therapy, and supported with diode laser LLLT applications, which satisfactorily and safely serve as a complementary method to conventional medical and/or surgical interventions. Similarly, Guarda et al. reported positive effect on healing of a BRONJ lesion and remission of painful symptoms with Ga-Al-As laser biostimulation [30]. They reported statistically significant difference in pre- and postoperative scores of parameters such as edema, fistula, and pus formation when laser biostimulation was used. Romeo et al. performed LLLT in 12 BRONJ cases every 3 days for 2 weeks and reported that six patients showed significant pain reduction, and only one patient indicated a worsening of the symptoms, which was attributed to a reinfection on the site, while all patients showed clinical improvement [31]. Scoletta et al. applied LLLT with diode laser without debridement or conservative surgery [32]. Their results showed that the use of LLLT in the management of ONJ-BP had a significant effect on reducing pain and clinical parameters of inflammation. In an animal study by Sarkarat et al., LLLT was shown to reduce the inflammation, bone exposure, and BRONJ stage, by improving bone formation in extraction sockets of rats under BP therapy [33]. In contrast to above-mentioned promising results with LLLT, Favia et al. reported that monthly LLLT administration without surgical intervention did not reveal successful outcomes and 87.5% of the lesions remained stable [34]. Further, Atalay et al. reported that in ten patients undergoing laser surgery with Er:YAG laser and Nd:YAG biostimulation, there was no difference between laser surgery and conventional surgery groups [35].

Similar to LLLT therapy, ozone therapy has been claimed to have a positive effect on both soft tissues and bone through stimulation of endogenous antioxidants. Among previously described therapeutic properties of ozone therapy are antimicrobial effect against anaerobic and aerobic bacteria, fungi, and viruses, stimulation of hemoglobin and red blood cell production with a relative increase in blood oxygenation, regulation of cytokines involved in the immune response, increased phagocytosis and diapedesis, and stimulation of angiogenesis and fibroblasts [36–38].

Some of the previous studies investigating ozone therapy and MRONJ have shown that this technique can stimulate cell proliferation and soft tissue healing [39–41]. Petrucci et al. administered preoperative and postoperative ozone in patients undergoing surgical treatment for MRONJ who presented with pain, secretion, and halitosis and reported reduction in all symptoms [42]. Agrillo et al. performed pre- and postoperative ozone supported surgical debridement in 33 MRONJ cases and reported that 54% of the patients showed complete healing, while 30% of them experienced significant reduction in their symptoms [43]. Similarly, in another retrospective study by Agrillo et al., it has been reported that 91% of 131 patients affected by MRONJ had improvement by adjunctive use of ozone [36]. Goker et al. also proposed an alternative method for ozone application in which the ozone/oxygen mixture was applied with local submucosal infiltrations around the necrotic area and in fistulous tracts [44]. The mixture was applied twice a week for 10 weeks (5 weeks before surgical debridement and 5 weeks postoperatively) and the authors reported an overall success rate of 64.2% with this method. In addition to these clinical applications, Monteiro et al. administered ozonated oil to the rats for 10 min/day during 3 days after upper 1st molar extraction and reported that ozonated oil may reduce the development of osteonecrosis [13].

Apart from these studies, Kazancioglu et al. investigated and compare the effects of ozone therapy and LLLT on bone formation during the process of bone healing and demonstrated that both ozone and laser therapies had a positive effect on bone formation in healthy rat calvarial defect compared with the control group; however, in contrast to our findings, ozone therapy was found more effective than LLLT [45].

The present study revealed results, which confirm findings of several previous studies, indicating positive effects of LLLT and ozone in the prevention of MRONJ. However, it is important to bear in mind that this study was set out in an experimental design, which excluded multiple variables that can affect the occurrence or progress of MRONJ (such as concomitant diseases, longevity of BP therapy, oral hygiene, etc.) in a clinical scenario. Further research including randomized controlled clinical trials is necessary to confirm the results of the current study.

## Conclusions

Given the findings of the present study and current literature, adjunctive use of LLLT and gaseous ozone may be beneficial in prevention of MRONJ.

## Data Availability

The datasets used and/or analyzed during the current study are available from the corresponding author on reasonable request.
